# Signatures of Quantum Mechanics in Chaotic Systems

**DOI:** 10.3390/e21060618

**Published:** 2019-06-22

**Authors:** Kevin M. Short, Matthew A. Morena

**Affiliations:** 1 Integrated Applied Mathematics Program, Department of Mathematics and Statistics, University of New Hampshire, Durham, NH 03824, USA; 2 Department of Mathematics, Christopher Newport University, Newport News, VA 23606, USA

**Keywords:** quantum entanglement, chaotic entanglement, chaotic systems, cupolets, entropy, correspondence, unstable periodic orbits

## Abstract

We examine the quantum-classical correspondence from a classical perspective by discussing the potential for chaotic systems to support behaviors normally associated with quantum mechanical systems. Our main analytical tool is a chaotic system’s set of cupolets, which are highly-accurate stabilizations of its unstable periodic orbits. Our discussion is motivated by the bound or entangled states that we have recently detected between interacting chaotic systems, wherein pairs of cupolets are induced into a state of mutually-sustaining stabilization that can be maintained without external controls. This state is known as chaotic entanglement as it has been shown to exhibit several properties consistent with quantum entanglement. For instance, should the interaction be disturbed, the chaotic entanglement would then be broken. In this paper, we further describe chaotic entanglement and go on to address the capacity for chaotic systems to exhibit other characteristics that are conventionally associated with quantum mechanics, namely analogs to wave function collapse, various entropy definitions, the superposition of states, and the measurement problem. In doing so, we argue that these characteristics need not be regarded exclusively as quantum mechanical. We also discuss several characteristics of quantum systems that are not fully compatible with chaotic entanglement and that make quantum entanglement unique.

## 1. Introduction

Chaotic behavior is generally attributed to a sensitive dependence on initial conditions and is characterized by a positive maximal Lyapunov exponent that causes nearby trajectories to diverge from each other exponentially fast. Despite its ubiquity in classical physics, chaos is yet to be rigorously established within quantum settings. One explanation for this disparity is that, unlike chaotic or classical systems, whose states may be completely described by a set of dynamical variables, in quantum mechanics, conjugate observables such as position and momentum cannot take on well-defined values at the same time. Particle dynamics are instead determined in part by the uncertainity principle and by the linearity of the Schrödinger equation, which preserves the overlap between quantum states. In other words, the nonlinearity required for chaotic dynamics and the exponential divergence of neighboring trajectories are fundamentally incompatible with quantum mechanics in its present formulation.

In addition, however, much effort has recently been devoted to detecting signatures of chaos in quantum systems [1,2,3,4,5]. One such signature is the sensitivity of some quantum systems to perturbation. This has been experimentally observed in the decay in the overlap between quantum states that are evolving under slightly different Hamiltonians and is attributed to the positivity of a classically-derived Lyapunov exponent [1,4]. In fact, the rate of overlap decay is known to transpire at different rates depending on whether the evolution begins from initial conditions that correspond classically to chaotic versus regular regimes [5]. A second signature is quantum scarring, which refers to the scenario in which a quantum system’s associated wave function concentrates on paths that represent periodic orbits in the classical limit [6,7]. This phenomenon has been experimentally observed in several recent studies [8,9].

Entanglement in the purely quantum sense has also been observed to be a reliable indicator of classical chaos [10,11,12,13]. In Chaudhury et al.’s recent kicked top experiments of laser-cooled Cesium (${}^{133}$Cs) atoms, each atom’s initial state is followed for several periods of the “kicked” Hamiltonian, and the corresponding classical phase space reveals islands of regular motion surrounded by a sea of chaos [5]. When entropy is used to measure the entanglement, stronger entanglement is detected between entangled atoms whose states are initially prepared from chaotic regimes, whereas weaker entanglement is measured between atoms that evolve from regular regions. It is as if the quantum regime respects an underlying classical presence [13].

One feature of chaotic systems typically encountered in investigations is the infinite set of unstable periodic orbits (UPOs) that are found densely embedded in associated attractors. These orbits collectively provide a rich source of qualitative information about the parent chaotic system and are the focus of numerous theoretical and practical applications [14,15,16]. As a result, several control schemes have been designed to detect and stabilize these orbits [17,18,19]. In Section 2 of this paper, we discuss an adaptation of one particular control method that very efficiently stabilizes the *cupolets* of chaotic systems (*C*haotic, *U*nstable, *P*eriodic, *O*rbit-*LETS*) [20,21,22,23].

Cupolets are controlled and stabilized periodic orbits of a chaotic system that would normally be unstable without the presence of the control mechanism. These orbits represent approximations to the UPOs but are distinguished because their stabilization supports a one-to-one correspondence between a given sequence of controls and a specific cupolet, with each cupolet able to be generated independently of initial condition. In Section 4.2, we derive the functional form of a cupolet, which further establishes the suitability of cupolets for analyzing chaotic systems.

In recent studies, we report on the proclivity for chaotic systems to enter into bound or entangled states [23,24,25]. We demonstrate how pairs of interacting cupolets may be induced into a state of mutually-sustaining stabilization that requires no external controls in order to be maintained. This state is known as *chaotic entanglement*, and it is self-perpetuating within the cupolet-stabilizing control scheme, meaning that each cupolet of an entangled pair is effectively controlling the stability of its partner cupolet via their continued interaction. The controls used are all information-theoretic, so we stress that additional work is required to relate this research directly to physical systems. However, many of our simulated cupolet-to-cupolet interactions are based on the dynamics of physical systems, and so our findings signal the potential of chaotic entanglement to be both physically realizable and naturally occurring. It is worth noting the sensitivity of chaotic entanglement to disturbance since any disruption to the stability of either cupolet of an entangled pair may be enough to destroy the entanglement. It is this property in particular that establishes chaotic entanglement as a classical analog of quantum entanglement [25]. It is also worth noting that chaotic entanglement is an entropy-reversing event, according to the calculation of entropy as the rate in which dynamical systems generate information over time.

We are aware that entanglement is regarded as a quantum phenomenon and that there are characteristics of quantum entanglement that are incompatible with chaotic entanglement, such as nonlocality. We are also aware that chaotic entanglement has been previously examined in [26], while a classical version of entanglement has been proposed in [27]. In the first study, linear and nonlinear subsystems are coupled together to produce composite chaotic systems, a synthesis the authors refer to as chaotic entanglement. In the second study, a classical version of quantum entanglement is demonstrated via a beam of photons and is shown to be consistent with many features of quantum entanglement, apart from nonlocality. In contrast, the novelty of the chaotic entanglement that we have documented arises in how two interacting chaotic systems are induced into a state of mutual stabilization. First, the chaotic behavior of the two systems is collapsed onto unique periodic orbits (cupolets). Following the collapse, the ensuing periodicity of each chaotic system and the stability of each cupolet are maintained intrinsically by each system’s dynamical behavior and will persist until the interaction is disturbed. To our knowledge, this is the first documentation of chaotic systems interacting to such an extent.

Our initial results are very promising given that we have identified hundreds of pairs of entangled cupolets from several low-dimensional chaotic systems [25]. When regarded as a parallel to quantum entanglement, chaotic entanglement is further intriguing because it demonstrates that chaotic systems are capable of exhibiting behavior that has conventionally been associated exclusively with quantum systems. Accordingly, we shall now discuss the potential for chaotic, classical systems to support additional parallels with quantum mechanics, namely the measurement problem, Hilbert space constructions, notions of wave function collapse, superposition of states, and various entropy definitions.

Our discussion uses cupolets and chaotic entanglement as reference points and is organized as follows. In Section 2, we begin by providing a brief introduction to cupolets and how they are generated, and then we discuss a few of their interesting properties and applications. In Section 3, we describe chaotic entanglement and how it can be induced and detected between pairs of interacting chaotic systems. The main discussion of chaotic systems exhibiting quantum-like behavior is found in Section 4. Finally, we offer a few concluding remarks in Section 5.

## 2. Background on Cupolets

Broadly speaking, cupolets are a relatively new class of waveforms that were originally detected while controlling a chaotic system in a secure communication application. The theory behind these orbits and their applications has been well-documented [15,16,20,21,22,23,24,25,28,29]. In this section, we summarize first the control technique that is used to generate cupolets and then the applications of cupolets that have particular relevance to our chaotic entanglement research. More technical details of the control process can be found in [22,23,25,28,29].

### 2.1. Generating Cupolets

The control scheme that is used to stabilize cupolets is adapted from one designed by Hayes, Grebogi, and Ott (HGO) [18,19]. In the HGO scheme, small perturbations are used to steer trajectories of the double scroll system, also known as Chua’s oscillator, around an attractor. The differential equations describing this system are given by:

(1a) $$\begin{array}{cc} {\dot{v}}_{C_{1}} & =\frac{G\left(v_{C_{2}}-v_{C_{1}}\right)-g\left(v_{C_{1}}\right)}{C_{1}},\\ {\dot{v}}_{C_{2}} & =\frac{G\left(v_{C_{1}}-v_{C_{2}}\right)+i_{L}}{C_{2}},\\ {\dot{i}}_{L} & =-\frac{v_{C_{2}}}{L}, \end{array}$$

where the piecewise linear function, g(v), is given by:

(1b) $$\begin{array}{cc} g(v) & =\left\{\begin{array}{cc} m_{1}v, & \mathrm{if}\;|v|\leq B_{p},\\ m_{0}\left(v+B_{p}\right)-m_{1}B_{p}, & \mathrm{if}\;v\leq -B_{p},\\ m_{0}\left(v-B_{p}\right)+m_{1}B_{p}, & \mathrm{if}\;v\geq B_{p}. \end{array}\right. \end{array}$$

**Figure 1:**
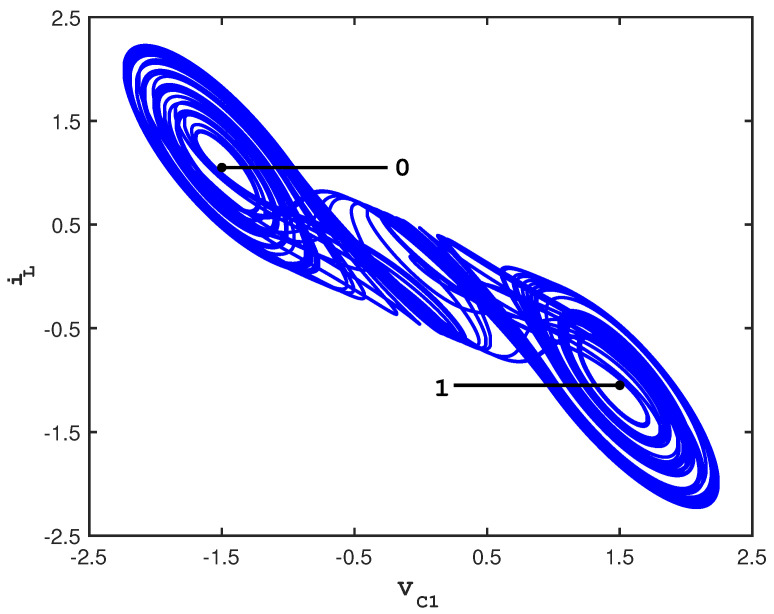
2D projection of the double scroll attractor showing the control surfaces.

When $C_{1}=\frac{1}{9}$, $C_{2}=1$, $L=\frac{1}{7}$, G=0.7, $m_{0}=-0.5$, $m_{1}=-0.8$, and $B_{p}=1$, the double scroll system is known to be chaotic, and its attractor consists of two lobes that each surrounds an unstable fixed point [30]. Figure 1 shows a typical trajectory tracing out this attractor.

Control of the double scroll system is achieved by setting up two control planes on the attractor (via a Poincaré surface of section) and by partitioning each control plane into *N*-many small control bins. The control planes are assigned binary values so that a binary symbolic sequence may be recorded whenever a trajectory intersects a control plane. This particular symbolic sequence is known as a *visitation sequence*. Perturbations are applied only when a trajectory evolves through the control bins; otherwise, the trajectory is allowed to evolve freely around the attractor. Every time the trajectory intersects a control plane, *microcontrol* perturbations reset the trajectory to the center of the control bin through which it passes. In some instances, *macrocontrols* are also applied. Macrocontrol perturbations are specifically defined via the HGO technique to be the smallest perturbation along a control plane necessary to produce a change of lobe *N*-many loops downstream in the visitation sequence. In this way, a chaotic system can be directed to follow a prescribed visitation sequence. Figure 1 also shows the positions of these control planes which emanate outward from the center of each lobe. We typically partition each control plane into 2000-many bins, so that the ensuing perturbations are guaranteed to be small.

**Figure 2:**
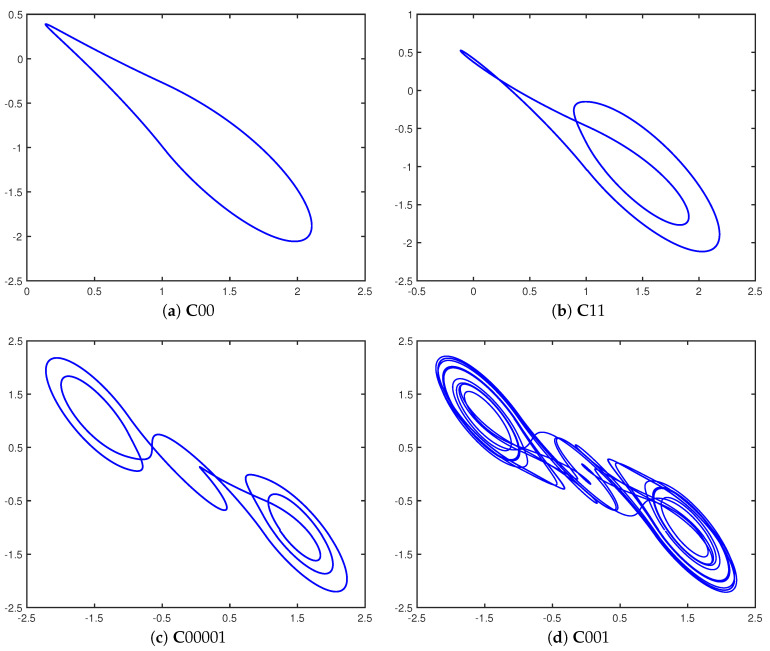
Cupolets of various periods belonging to the double scroll system. The control sequences that must be periodically applied in order to stabilize these periodic orbits are (**a**) ‘00’, (**b**) ‘11’, (**c**) ‘00001’, and (**d**) ‘001’.

Parker and Short [20] later combined this control scheme with ideas from the study of impulsive differential equations [31] and discovered that when a repeating binary *control sequence* is used to define the controls, with a ‘1’ bit corresponding to a macrontrol perturbation and a ‘0’ bit corresponding to only a micontrol perturbation, then the double scroll system stabilizes onto a periodic orbit. In fact, the stabilization occurs regardless of the current state of the system or of the starting bit of each repeating control sequence. These periodic orbits have been given the name *cupolets*, and this work has since been extended to chaotic maps and a variety of other continuous chaotic systems such as the Lorenz and Rössler systems. The examples of double scroll cupolets appearing in Figure 2 are generated by repetitively applying the indicated control sequences to the double scroll system.

### 2.2. Cupolet Properties and Stability

To summarize, cupolets are highly-accurate approximations to the UPOs of chaotic systems that are generated by adaptating the HGO control technique accordingly. Cupolets exhibit the interesting properties of being stabilized independently of initial condition and also of being in one-to-one correspondence with the control sequences. These controls can be made arbitrarily small and thus do not grossly alter the topology of the orbits on the chaotic attractor. This suggests that cupolets are shadowing true periodic orbits, and theorems have been developed to establish conditions under which this holds [22,32,33,34,35]. What further distinguishes cupolets from UPOs, which are traditionally stabilized via techniques such as Newton’s or first-return algorithms, is that large numbers of cupolets can be inexpensively generated by just a few bits of binary control information. For example, over 8800 double scroll cupolets can be stabilized from implementing 16-bit or fewer control sequences.

For a given cupolet to remain stabilized, all that is required is the repeated application of its control sequence to the system. Applying different controls would induce the system to destabilize from a stabilized cupolet and revert to chaotic behavior. If a second sequence of controls were to then be periodically applied, the chaotic system would eventually restabilize onto a second cupolet, possibly after some intermediary transient phase. Any transient is the result of the trajectory evolving while the chaotic system sifts through all possible states until it reaches one where the behavior of an UPO falls into synchrony with the control sequence, thus stabilizing the cupolet. Cupolet restabilization is guaranteed because of the injective relationship that exists between cupolets and the binary control sequences. This makes it possible to transition between cupolets, and thus between UPOs, simply by switching control sequences [29].

## 3. Chaotic Entanglement

In previous work [23,24,25], we document the surprising observation that pairs of chaotic systems may interact in such a way that they *chaotically entangle*. To do so, two chaotic systems must first induce each other to collapse and stabilize onto a cupolet (e.g., periodic orbit) via the exchange of control information. The stabilities of the two stabilized cupolets must also become deterministically linked: disturbing one cupolet from its periodic orbit subsequently affects the stability of the partner cupolet, and vice versa. Hundreds of entangled cupolet pairs have been identified for the double scroll system, and it has been shown that chaotic entanglement evokes several connections to quantum entanglement, as we discuss in Section 3.2 below.

Cupolets from two entangled chaotic systems are regarded as mutually stabilizing because their interaction essentially serves as a two-way coupling that is self-perpetuatating within the control scheme described in Section 2. Once entanglement has been established between two chaotic systems, no outside intervention or user-defined controls are needed in order to maintain the stabilities of their respective cupolets. The stability of each cupolet is instead preserved by the dynamics of the partner cupolet. Not only has the original chaotic behavior of the two parent systems collapsed onto the periodic orbits of the two cupolets, but this periodic behavior will persist as long as their interaction is undisturbed.

Chaotic entanglement is typically mediated by an *exchange function* that defines the interaction between the two chaotic systems and their cupolets. Exchange functions are described more fully in [25] as catalysts for the entanglement and are taken to represent the environment or medium in which the interacting systems are found. For instance, several types of exchange functions have been designed that simulate the interactions of various physical systems, such as the integrate-and-fire dynamics of laser systems and networks of neurons. These exchange functions have all successfully induced chaotic entanglement in the double scroll system.

### 3.1. Chaotic Entanglement through Periodic Orbits

In Section 2, we defined a cupolet’s visitation sequence to be the binary sequence of lobes that its orbit visits. Visitation sequences thus serve as a type of symbolic dynamics for chaotic systems; i.e., dynamic information that is generated as solutions to these systems evolve over time. With this in mind, chaotic entanglement is more technically characterized as an exchange of symbolic information in the form of visitation sequences.

In order for a pair of interacting chaotic systems to chaotically entangle, the easiest way to view it is that one of the systems must first be externally stabilized onto a cupolet, say $C_{A}$. As $C_{A}$ evolves about its attractor, the bits of its visitation sequence are passed to an exchange function which then performs a binary operation on the visitation sequence. The outputted sequence of bits is known as an *emitted sequence* and is taken as a control sequence and applied to the other chaotic system. This induces the second system to stabilize onto a second cupolet, say $C_{B}$. Concurrently, but in the reverse direction, the visitation sequence belonging to $C_{B}$ passes through the same exchange function, and the resulting emitted sequence is applied as control instructions to $C_{A}$. At this point, the cupolets of the two parent systems are both receiving and transmitting control information via the exchange function, but if the emitted sequence generated from the visitation sequence of $C_{A}$ matches the control sequence needed to maintain the stability of cupolet $C_{B}$—and vice versa—then the two parent chaotic systems, via their cupolets, have become intertwined in a mutually-stabilizing feedback loop and are considered chaotically entangled. The external controlling can be discontinued now that each cupolet’s visitation sequence is preserving the partner cupolet’s stabilization.

**Figure 3:**
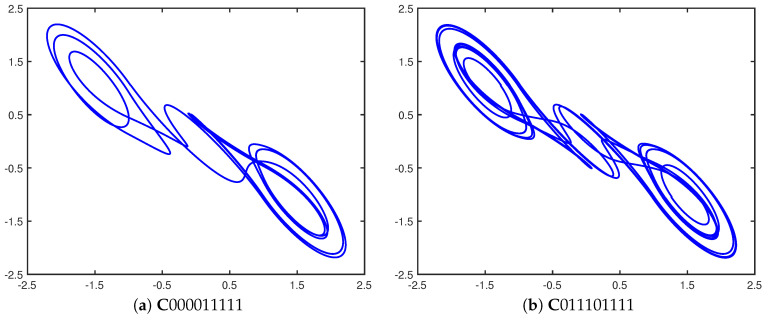
Entangled cupolets (**a**) C000011111 (period 9) and (**b**) C011101111 (period 18). The visitation sequences of these cupolets are V000111111 and V000000000111111111, respectively.

**Figure 4:**
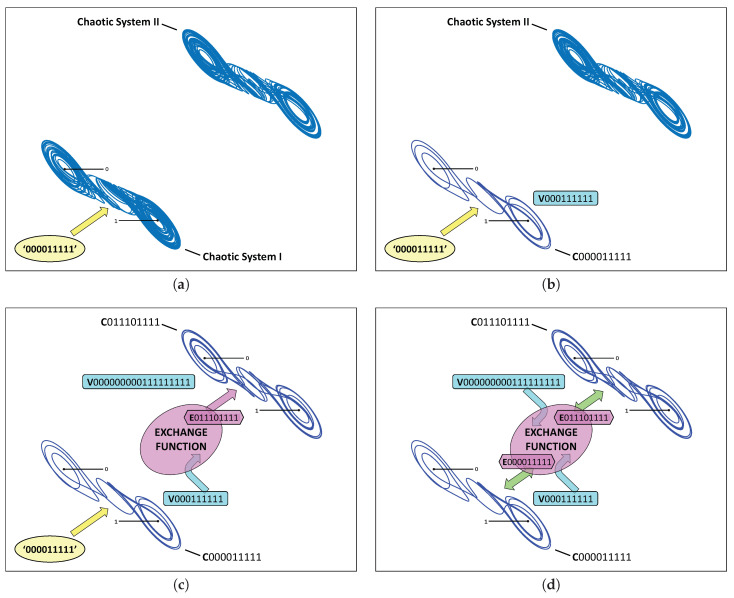
(Color online) Schematic illustration of chaotic entanglement: in (**a**) a control sequence is externally applied to Chaotic System I, via the indicated (yellow) control pump. System I subsequently stabilizes in (**b**) onto cupolet C000011111 according to the control method described in Section 2. This cupolet then evolves around the attractor to generate its visitation sequence, V000111111. In (**c**), an exchange function accepts this visitation sequence as an input and the outputted emitted sequence, E011101111, is taken as a control sequence and used to control a second chaotic system, System II. System II subsequently stabilizes uniquely onto cupolet C011101111 whose visitation sequence, V000000000111111111, is then passed to the same exchange function in (**d**). The resulting emitted sequence, E000011111, is applied as control instructions to C000011111 of System I. Note that each emitted sequence exactly matches each corresponding cupolet’s control sequence, and so the external control pumps seen in (**a**–**c**) are unnecessary and can be removed. Systems I and II are now dynamically engaged in a state of perpetual mutual-stabilization between their respective cupolets and are thus considered chaotically entangled. This entanglement is summarized in Table 1.

As an example, we will now demonstrate how the two cupolets shown in Figure 3 can become chaotically entangled. This process is also depicted in Figure 4 as a series of step-by-step illustrations. First, two chaotic double scroll systems, Systems I and II, are simulated without control. In order to stabilize one of these cupolets, say C000011111, the control sequence ‘000011111’ must be applied to System I using the cupolet-generating control technique described in Section 2. This step is illustrated in Figure 4a, where the depiction of the control planes indicates that Chaotic System I is being controlled via the (yellow) external control pump. Once C000011111 completes one full period around the attractor, its visitation sequence, V000111111, is realized. Figure 4b captures this stage of the entanglement process. This visitation sequence is then passed to an exchange function where it is modified according to a predefined binary operation and transmitted to System II as an emitted sequence. In this example, a ‘preponderance’ exchange function converts V000111111 into the emitted sequence E011101111 essentially by interpreting the bits of a visitation sequence as binary energy and imposing an energy threshold on the visitation sequence. This particular type of exchange function is described more thoroughly in [25]. As the emitted sequence passes from the exchange function, it is applied to System II as instructions for controlling this system. In this case, System II stabilizes onto the cupolet C011101111 since this emitted sequence actually is this second cupolet’s control sequence. These particular steps are depicted in Figure 4c.

The cupolets’ interaction now repeats in the reverse direction. The visitation sequence of cupolet C011101111 is found to be V000000000111111111 (see Figure 4c), which is converted by the same exchange function into the emitted sequence E000011111. When applied as a control sequence to System I, E000011111 sustains the stability of cupolet C000011111 because the emitted bits, ‘000011111’, match the control information needed to maintain this cupolet’s stability. This two-way exchange of control information between Systems I and II defines the cupolets’ interaction, which has been managed by the exchange function. Notice that both emitted sequences, E011101111 and E000011111, match the required control sequences for cupolets C000011111 and C011101111, respectively. The (yellow) external control pump is thus redundant and can be discarded now that the cupolets are themselves dynamically generating the necessary control instructions. Since the cupolets are driving each other’s stability, they are considered chaotically entangled, and their stabilities are guaranteed so long as their two-way interaction is undisturbed. Figure 4d illustrates this final step of the entanglement process, while Table 1 summarizes the correspondence between the control, visitation, and emitted sequences of each cupolet.

Strictly speaking, chaotic entanglement need not be associated exclusively with interacting cupolets because its generation extends naturally to pairs of interacting UPOs. Cupolets represent highly accurate approximations to these UPOs, but in general two interacting chaotic systems will chaotically entangle once each system stablizes onto a particular UPO whose stability is then maintained by the symbolic dynamics of the partner UPO. The visitation sequences of the UPOs would continue to provide an appropriate symbolic dynamics, but the advantage of inducing and detecting entanglement with cupolets is twofold.

First, the control technique described in Section 2 is designed to stabilize cupolets. In doing so, the technique makes accessible the symbolic dynamics of chaotic systems while greatly simplifying how the interactions between the systems are simulated. In particular, perturbations are applied only when a cupolet intersects a control plane, which means that the cupolet’s remaining evolution is freely determined by the system’s governing equations. Therefore, when detecting entanglement between two systems, one only needs to monitor finitely-many intersections with the control planes, which in turn allows one to simultaneously observe the visitation sequence of each evolving cupolet. Second, given that cupolets can be generated very efficiently, a great deal of useful information can be collected simply by preemptively recording the control and visitation sequences of a sufficiently large collection of pre-generated cupolets. This has been shown to facilitate the detection of cupolets that could potentially chaotically entangle with a given cupolet [25].

### 3.2. Chaotic Entanglement as an Analog of Quantum Entanglement

Chaotic entanglement exhibits many properties that are characteristic of quantum entanglement. For instance, measurements that disrupt the interaction between two entangled cupolets, say $C_{A}$ and $C_{B}$, will almost always destroy their entanglement unless a great deal is known about the control scheme. By measurement, we mean a perturbation that could be as meticulous as the microcontrols or macrocontrols that are implemented by the aforementioned control scheme, or as general as an arbitrary perturbation applied to one of the two parent systems. As an example, consider the subtle effect of interchanging a ‘0’ bit for a ‘1’ bit in the control sequence of $C_{A}$. Control sequences are unique since they direct a chaotic system onto one specific cupolet. Disturbing the cupolet’s control sequence would perturb its trajectory into a different bin on the control plane, causing $C_{A}$ to destabilize from its periodic orbit. In this scenario, $C_{A}$ would produce a different visitation sequence that no longer guarantees the stability of the partner cupolet $C_{B}$, and so their entangled state would be lost. However, should the appropriate controls for cupolet $C_{A}$ be restored and continue to be periodically applied, then $C_{A}$ and $C_{B}$ would eventually restabilize via the restarted entanglement process.

In Section 4, we describe how a measurement can be carefully designed so that its effects are not significant enough disturb the stability of the intended cupolet. Implementing these sorts of measurements would allow one to probe a pair of entangled cupolets without compromising their entanglement and would also provide access to the control sequences that are associated uniquely to each cupolet. In other words, having full knowledge of the control mechanism would permit the control information stored in an entangled state to be recovered and read later. In this way, entangled cupolets remember the state of the control bits that are originally used in establishing their entanglement and naturally form a memory device for information. This process of inserting, storing, and retrieving information in pairs of entangled cupolets is similar to what is currently being developed with quantum computing [2,36].

### 3.3. Pure Chaotic Entanglement

In some instances, chaotic entanglement occurs without the assistance of an exchange function (or, equivalently, via an identity exchange function). This is known as *pure entanglement* because it requires no environmental property in order to be induced or sustained [23,25]. Instead, a visitation sequence is converted directly to an emitted sequence without any intermediary modification being made. That is, each purely-entangled cupolet generates the exact sequence of control bits necessary for maintaining its partner’s periodic orbit without any assistance from an exchange function, but simply by realizing its own visitation sequence. This makes pure entanglement the simplest form of cupolet entanglement. Entanglement induced with the aid of an exchange function is considered a variation of pure entanglement because an environmental effect or a nontrivial operation must be performed on a cupolet’s visitation sequence in order to generate an emitted sequence.

Existence of pure chaotic entanglement has been documented in [23,25] and indicates the potential for such behavior to arise naturally between interacting chaotic physical systems; i.e., independent of the external controls. With no external controller or exchange function needed as a catalyst, it is possible that such direct cupolet-to-cupolet interactions may arise naturally and lead to naturally entangled states. This may not be altogether surprising given that experimental evidence of natural and macroscopic quantum entanglement has recently been reported in [37,38,39]. Spontaneous chaotic entanglement is further discussed in Section 4 when we consider additional connections between chaotic and quantum systems.

## 4. Main Discussion: Parallels between Chaotic and Quantum Systems

Chaotic entanglement demonstrates a new way for chaotic systems to interact and thus signals a new parallel between quantum and classical mechanics. We now further explore this connection by discussing other properties of chaotic systems that quantum systems also exhibit. We also address several concerns that invariably arise when examining entangled quantum systems and how these concerns relate to chaotic systems. Key to our discussion is the important role that the cupolets and UPOs of chaotic systems play in determining the dynamical properties of chaotic systems.

### 4.1. Hilbert Space Considerations

Formulating a Hilbert space of states is taken as a starting point in many quantum studies. This allows one to express an associated wave function as a linear combination of orthonormal state vectors that satisfies the Schrödinger equation. For instance, one way to formulate the Hilbert space of a quantum system is via the Fourier modes of the system: one can assemble linear combinations of sinusoids in order to define the state vectors as is done with the infinite square well [40].

Constructing a Hilbert space on a chaotic system is not as straightforward because the governing equations are nonlinear and prevent linear combinations of states from also being solutions. Cupolets are highly-accurate approximations to the periodic solutions of chaotic systems, and so one could designate cupolets (e.g., UPOs) as the state vectors, except that cupolets and UPOs do not satisfy any simple orthogonality principles. Moreover, chaotic systems generally admit a countably infinite number of these periodic orbits on their attractors [41], and so cupolets and UPOs would form an overdetermined set of basis elements. In fact, the Fourier spectra obtained from any large collection of cupolets is also overdetermined: although the simplest cupolets consist of just one or two spectral peaks, the higher period cupolets exhibit tens or hundreds of significant peaks in their spectra [15,16].

However, cupolets and UPOs are still regarded as the states of chaotic systems, even if superpositions of these orbits are unable to satisfy the underlying equations. This is because ergodicity guarantees that a free-running chaotic system ultimately realizes all possible non-equilibrium states and visits arbitrarily small neighborhoods of its periodic solutions infinitely often. Even though chaotic systems evolve aperiodically for all time, the dynamics of these systems are ultimately confined to their attractors, which means that a wandering chaotic trajectory undergoes a series of close encounters with the embedded UPOs and cupolets.

Furthermore, many characteristics of chaotic systems, such as Lyapunov exponents, the natural measure, dimension, topological entropy, and several orbit expansions, can all be expressed in terms of UPOs [14,17,42,43,44]. In particular, the natural measure, which is roughly interpreted as the probability of a chaotic system visiting a given region of its attactor over time, is often described as being concentrated on the UPOs. In other words, as it evolves, a chaotic system visits regions populated by UPOs with greater frequency and will dwell alongside an UPO for an extended amount of time after which the trajectory begins shadowing other UPOs. Since UPOs are solutions to the governing differential equations, uniqueness properties imply that these orbits cannot be crossed in phase space. This is easier to visualize in many low-dimensional chaotic systems, such as the double scroll, Lorenz, and Rössler systems, where the attractors are locally ribbon-like in at least part of their domains. Cupolets are generated in such a way that uniqueness considerations also apply, except possibly at certain locations along a control plane where the controls are applied [29]. Chaotic trajectories are thus restricted to evolving along unique paths that are locally bounded by UPOs and cupolets, which means that the dynamics of chaotic systems are locally dependent on these orbits.

### 4.2. Functional Representation of Cupolets

While it is not straightforward to establish a superposition of Hilbert space basis elements for nonlinear dynamical systems, the periodic nature of cupolets does allow for functional representations of these orbits to be derived and used as (approximate) solutions to the nonlinear differential equations. This results in a low complexity approximation of the UPO solutions of the nonlinear differential equations. To demonstrate this, we will now derive the functional form of two cupolets and then show how well the functional form approximates the corresponding true periodic orbits obtained through numerical integration.

Since cupolets are periodic over the attractor, they play the role of eigenfunctions for the differential equations, and because of their periodicity, the Fourier decompositions of cupolets converge rapidly. Hence, one can use the Fourier representation of a cupolet—itself a finite dimensional expansion over a discrete Hilbert space—to create a functional form that can be used in symbolic computational systems like Mathematica (Version 11.3, Wolfram Research: Champaign, IL, USA). Furthermore, one can look at the fast Fourier transform (FFT) of sampled cupolet data to determine which Fourier coefficients are significant, and then truncate the representation so that only the significant Fourier modes are retained. As we demonstrate below, the functional form of a cupolet compares favorably to its numerical solution which is obtained directly from the uncontrolled differential equations of the double scroll system.

In order to create a functional representation of a cupolet, the time domain data from the numerical simulation of the cupolet is preemptively stored in a vector of 1024 samples. A cupolet’s period, *T*, in simulated time varies among the cupolets, and this value needs to be initially recorded as well. The numerical integration of the system must be carefully managed so as to maintain accurate time steps even as the system passes through the control planes and is subjected to the perturbations of the control scheme described in Section 2 [22]. Even so, the cupolets are often extremely close to the true UPOs of the system. The vector of samples is then passed through the FFT to create a vector of P=1024 frequency components. Of these components, one is a constant term, 511 are designated as “positive spinning” components, 511 are designated as “negative spinning” components, and one is associated with the Nyquist frequency that is neither positive- nor negative-spinning. The term “negative spinning” simply means that the complex sinusoids are sampled by moving in the negative angle (e.g., clockwise direction) around the complex unit circle.

The derivation of the functional representation of a cupolet proceeds as follows. We let the vector of 1024 samples of the cupolet be represented as $\vec{s}$, with individual entries designated $s_{k}$, and the corresponding frequency domain coefficients as $C_{f}$. The initial FFT is calculated as

(2) $$C_{f}=\sum_{k=0}^{P-1}W^{\left(k*f\right)}\;s_{k},$$

where $W=e^{i2\pi /P}$ [45]. Note that the Nyquist frequency coefficient is $C_{P/2}$. It is now useful to relabel the *f* index because half of these coefficients are negative and reflect the “negative spinning” aspect of the complex sinusoids. The relabeling is done for all indices f>P/2, in which case f→f−P. Now that the negative indices represent negative spinning oscillators, they can be grouped with the correspondingly-labeled positive spinning oscillators in complex conjugate pairs. Under this relabeling, the original sampled values can each be recovered exactly from Equation (2) via the inverse FFT calculation:

(3) $$s_{k}=\frac{1}{P}\;\sum_{f=-\frac{P}{2}+1}^{P/2}W^{-k*f}\;C_{f}.$$

It is the inverse form of the FFT that allows for the conversion from discrete form to functional form, since each index *f* corresponds to an integer period complex sinusoid taken over the cupolet period *T*. Each *W* term in the sum corresponds to a discretely-sampled complex exponential, which has now been converted to a continuous-time complex exponential function. If we let τ be a placeholder for the continuous time component, we can take advantage of the complex conjugate pairing of the complex sinusoids and the corresponding coefficients $C_{f}$ and $C_{-f}$ in order to obtain a (real) functional form $C_{f}e^{i\tau }+C_{-f}e^{-i\tau }$, since the imaginary parts drop out. Next, we make explicit the integer period nature of the sinusoids and the period of the cupolet *T* by setting τ=2πft/T, where *t* represents continuous time. Consequently, we have defined the complex sinusoids to be periodic over a continuous time variable that naturally encodes both the period of the cupolet and the exact integer periods of the Fourier representation. This results in the (full) functional form of a given cupolet:

(4) $$s\left(t\right)=\frac{1}{P}\;\sum_{f=-\frac{P}{2}+1}^{P/2}\;C_{f}e^{i2\pi ft/T}.$$

Equation (4) can also be expressed in an equivalent form that shows the complex conjugate pairing along with the constant term and the (real) Nyquist term:

(5) $$s\left(t\right)=\frac{1}{P}C_{0}+\frac{1}{P}\;\sum_{f=1}^{P/2-1}\left[C_{f}e^{i2\pi ft/T}+C_{-f}e^{-i2\pi ft/T}\right]+\frac{1}{P}C_{\frac{P}{2}}\;e^{i2\pi Pt/2T}.$$

Once a cupolet’s functional form has been created, it can be used in a software package like Mathematica that allows for symbolic manipulation of mathematical equations. In addition, since many of the cupolets have rapidly decaying magnitudes for the Fourier/FFT coefficients, it is possible to keep only a subset of the coefficients in order to get a convenient functional form. In the examples presented below, we have P=1024, so there are 511 positive and negative frequency components (plus the constant and Nyquist terms). We can truncate the representation in Equation (4) to retain only *Q*-many components, where Q<P/2, giving

(6) $$s\left(t\right)=\frac{1}{P}C_{0}+\frac{1}{P}\;\sum_{f=1}^{Q}\left[C_{f}e^{i2\pi ft/T}+C_{-f}e^{-i2\pi ft/T}\right],$$

and in the examples below we will take Q=11 and Q=17.

**Figure 5:**
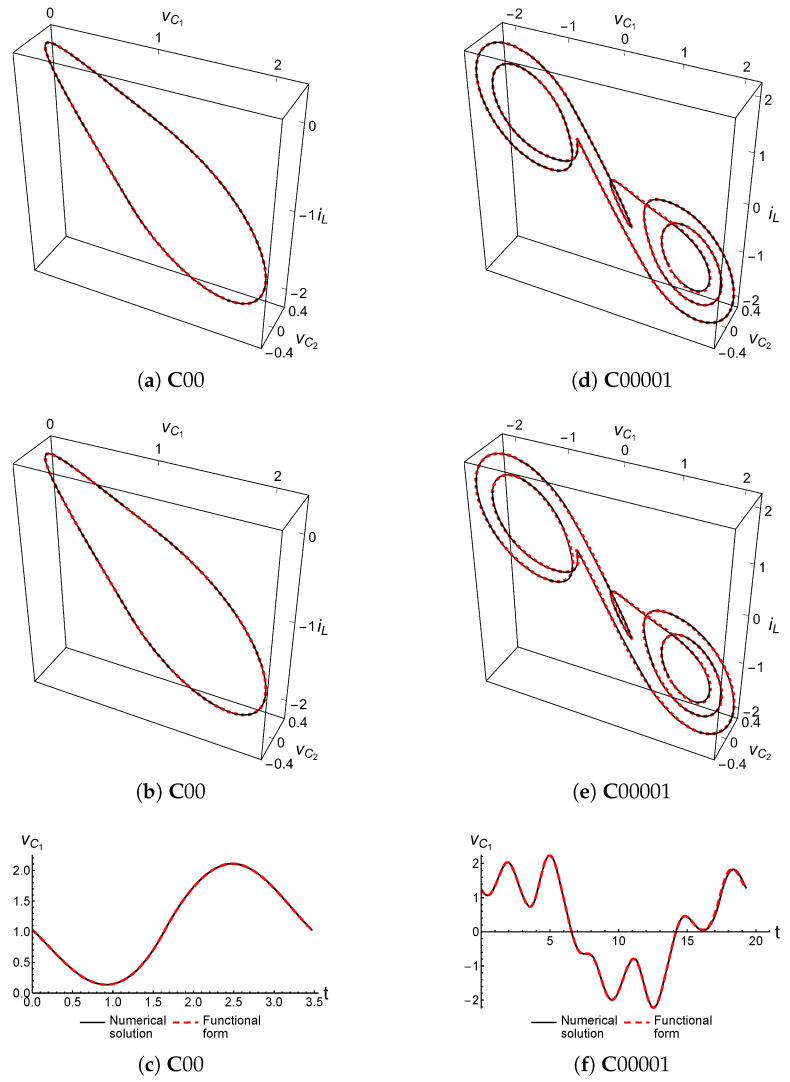
(Color online) Comparing the numerical solutions (solid black curves) of cupolets C00 (period 1) and C00001 (period 5) against their full and truncated functional representations (dashed red curves). In (**a**,**d**), the comparison is shown with the full version of the functional form (c.f., Equation (4)) for C00 and C00001, respectively. In (**b**,**e**), the comparison is shown with the truncated version using Q=11-many coefficients for C00 and Q=17 for C00001 (c.f., Equation (6)). The comparison between the $v_{C_{1}}$-component of the numerical solution and the corresponding truncated functional form is shown in (**c**) for C00 and in (**f**) for C00001. Cupolets are well-represented in both numerical and functional form, which is why the pairs of curves shown in each of these graphs are all effectively superimposed on each other. The simulated time periods of these cupolets are T≈3.46558 for C00 and T≈19.27239 for C00001.

To utilize this representation, the Mathematica software package can be used to compare the functional representation of the dynamical variables with the Mathematica numerical solution of the uncontrolled double scroll equations. Figure 5 shows the comparisons between the numerical solution and the full and truncated functional forms of two cupolets. The first cupolet is the simplest of all, cupolet C00, with the truncated version using Q=11 coefficients. The second example uses the 5-loop cupolet C00001, and the truncated version uses Q=17. In each case, the magnitude of the Fourier coefficients has diminished by over two orders of magnitude at the point where the series is truncated. Figure 5 also depicts the comparison between the $v_{C_{1}}$-component of the numerical solution and the corresponding truncated functional form for these cupolets. In all of these figures, the numerical data appear to be superimposed with the data obtained from the cupolets’ functional forms. This is because of how closely the functional forms approximate the cupolet’s true periodic orbit. Note that the orbits of these two cupolets have been seen previously in Figure 2.

While the cupolets provide useful approximations of UPO solutions of the nonlinear chaotic differential equations, there are still perturbations to a natural orbit from the controls applied on the control plane, so when there are larger perturbations, the fit to the uncontrolled solution will not be as close. Even so, a truncated cupolet expansion would provide a good starting point for a perturbative solution method, such as an improved Poincaré–Lindstedt solution, or a similar approach to developing a higher accuracy solution. In conclusion, while there is not a natural generic Hilbert space basis that satisfies the nonlinear dynamical equations of chaos, it is possible to use related techniques to develop functional approximations for the cupolets and UPOs that satisfy the constraint of being restricted to the chaotic attractor while also providing an approximate periodic solution.

### 4.3. Superposition of States

To represent the state of a given chaotic system as a superposition of cupolet states, let $\psi_{k}=\psi_{k}\left(t\right)$ denote the state space coordinates of the system’s $k^{th}$ cupolet at time t∈R, where k∈N. The state of the chaotic system, Ψ=Ψ(t), can then be expressed as a weighted sum of its cupolets:

(7) $$\Psi =\sum_{k=1}^{\infty }\alpha_{k}\psi_{k},$$

where each weight, $\alpha_{k}\in R$, represents the contribution to Ψ from cupolet $\psi_{k}$ at time *t* with respect to the natural measure c.f., [17,43]. As the chaotic system evolves in time, each $\alpha_{k}$ varies according to the proximity of the system to that cupolet. A chaotic system’s *state vector*, $\vec{\alpha }\in R^{\infty }$, is thereby formulated by collecting the weights of each cupolet:

(8) $$\vec{\alpha }=\left(\alpha_{1},\alpha_{2},\ldots ,\alpha_{k},\ldots \right).$$

The set of $\alpha_{k}$ will have local compact support because the cupolets that provide a nonzero contribution to the overall state of the system are those that are found within a local neighborhood of the current state of the system, whereas cupolets located farther away will contribute negligibly. In other words, when the system is dwelling near its *k*th cupolet, then $\Psi \approx \psi_{k}$ because at this moment $\alpha_{k}\neq 0$ and $\alpha_{l}\approx 0$ for all l≠k. Similarly, as the chaotic system deviates away from the *k*th cupolet, the dynamics are well approximated by nearby cupolets, say $\psi_{k-1}$, $\psi_{k}$, and $\psi_{k+1}$, while $\alpha_{l}\approx 0$ for more distant cupolets. To carry out an explicit calculation along these lines, there are several options. One can select a point on the attractor and then select segments of neighboring cupolets and use them to construct a model of the local dynamics [46,47], or one can adopt an approach like that of matching pursuit [48] and use the set of cupolets as a dictionary of states. Future work may compare a variety of methods of determining $\alpha_{k}$ for a set or subset of cupolets.

In quantum mechanics, the wave function is of fundamental importance since it provides a probabilistic description of the state of a quantum system. The analog for a chaotic system is its state vector, $\vec{\alpha }$, which provides a complete and evolving description for the state of a chaotic system in terms of its cupolets (or equivalently, its UPOs). In this way, a freely evolving chaotic system is viewed as evolving in a “mixed state” that is a superposition of cupolet states. In a mixed state, the contributions to the associated state vector come primarily from the cupolets in between which the chaotic trajectory is evolving and is nearest to at that moment.

### 4.4. Wave Function Collapse

Another fundamental concept in quantum mechanics is the idea that making a measurement induces the collapse of a quantum system’s associated wave function onto a specific state. Prior to the disturbance, the wave function is suspended in a superposition of state vectors, which inhibits the quantum system from being unambiguously described.

Similar behavior is supported by chaotic systems. When controls are repetitively applied to a chaotic system, cupolets form because of two key properties: the system stabilizes uniquely onto a periodic orbit under the influence of a set of repeating perturbations, and this stabilization occurs independently of initial conditions. These properties allow a chaotic system to be collapsed onto a specific cupolet from any initial state. The repeated action of the controls acts as the measurement process that induces wave function collapse. This occurs precisely when the chaotic system stabilizes onto a cupolet, say $\psi_{k}$. Via Equation (7), when this happens, $\alpha_{k}=1$ and $\alpha_{l}=0$ for all l≠k, which gives $\Psi =\psi_{k}$ as expected. The state vector given by Equation (8) reduces as well to $\vec{\alpha }=\left(0,\ldots ,0,1,0,\ldots \right)$, whose only nonzero component is its $k^{th}$. Until the collapse occurs, a chaotic system cannot be definitively described as a single cupolet state because it is instead locally dependent on a superposition of cupolet states.

It is important to stress that the interactions between chaotic systems that support chaotic entanglement would be such that the interaction could have the same effect as a measurement, so that the system in a chaotic state would collapse onto a periodic cupolet state. Thus, in chaotic entanglement, it may be fair to say that *interaction equals measurement*.

### 4.5. Natural Chaotic Entanglement

In chaotic systems, the concepts of measurement and state vector collapse are not only induced by external measurements or user-implemented controls, but are able to arise naturally in chaotic entanglement. Because their periodic orbits are unstable, isolated chaotic systems evolve aperiodically, yet a chaotic system tends to dwell significantly longer on its UPOs than on any other states or regions of phase space. By extension, an ensemble of independent chaotic systems would also each be dwelling along their UPOs and cupolets infinitely often. If one of these chaotic systems happens to dwell on a cupolet that exhibits the ability to entangle, and that can also communicate control information to a second nearby chaotic system, and if this interaction is as successful in the reverse direction, then the two interacting systems would entangle naturally.

In the context of two arbitrary cupolets, $\psi_{k}$ and $\psi_{l}$, this situation implies that the parent system of cupolet $\psi_{k}$ will approach and dwell on $\psi_{k}$ infinitely often. If a second chaotic system is at the same time dwelling near $\psi_{l}$, then entanglement would form naturally between the two systems, provided that the symbolic dynamics of the cupolets can be used to maintain their periodic behavior. In this way, isolated and independently-evolving chaotic systems would be perturbing each other with the interactions themselves playing the role of the controls or measurements. This makes it possible for entanglement to occur naturally, as has been emphasized both in Section 3.3 and in the recent studies of macroscopic systems examined in [37,38,39]. As we discuss below, the potential for natural chaotic entanglement plays a key role in the interpretation of making measurements on individual members of entangled cupolet pairs.

### 4.6. Measurement Problem

It is first worthwhile to compare the effects of a *knowledgeable measurement* on a chaotic system, as opposed to a *blind measurement*. For instance, if one has both knowledge of the control scheme and access to measurement tools that are smaller than the scale of the control bins, then one could monitor the state of a chaotic system without disturbing its trajectory. That is, one could design a measurement whose effects would not be strong enough to perturb an evolving cupolet to a new bin center on a control plane. The slight deviation from the original orbit could be small enough to be corrected the next time the cupolet intersects a control plane via the implementation of the microcontrols. This we define as a knowledgeable measurement because it permits one to not only study a cupolet without compromising its stability, but to also probe two entangled systems without compromising their entanglement.

If a measurement is not implemented as carefully, the repercussions would be more pronounced. Consider the effects of the measurement described earlier in Section 3.2, whereby a single ‘1’ control bit in a given cupolet’s control sequence is altered to a ‘0’ control bit. Such a disturbance would destabilize the cupolet and cause the parent system to either revert to chaotic behavior or to stabilize again after a potentially long transient period. This disturbance is known as a blind measurement, and it would cause the destablized orbit to begin generating a new visitation sequence. Had this cupolet been entangled with another cupolet, then the effects of the blind measurement would transfer to the partner cupolet by way of the exchange function. This is because the exchange function would begin producing a different emitted sequence that no longer matches the control sequence required to maintain the stability of the partner cupolet. The cupolets’ entanglement would then be lost.

Regarding the measurement problem, consider the situation in which a pair of cupolets has entangled, either through the deliberate preparation of an entangled state, or naturally through pure entanglement. If a knowledgeable measurement is conducted on one member of the entangled pair, then the state of the other member would be known with certainty (with the proviso that we have only found unique pairings at this point). Should the measurement process involve blind measurements, then the disrupted communication between the members of the entangled pair would induce the two parent systems to begin evolving independently. Similarly, if the interaction between members of an entangled pair is limited by distance, and if the entangled cupolets become too far separated, then their entanglement would decay as their communication wanes. This decay would not necessarily be very rapid, but would be determined by the local Lyapunov exponents of the two cupolets [14]. In these situations, the history of the previous entanglement would not be immediately erased because a measurement conducted on one member of the entangled pair would be predictive of the state of the second system, although the accuracy of the prediction would diminish over time.

In contrast, the principles of quantum mechanics dictate that making any measurement on a system immediately alters its state. This is problematic for researchers for whom knowing the actual state of a quantum system is important [49,50]. As indicated by Isham,

“… quantum theory encounters questions that need to be answered, one of the most important of which is what it means to say, and how it can be ensured that the individual systems on which the repeated measurements are to be made are all in the ‘same’ state immediately before the measurement. This crucial problem of *state preparation* is closely related to the idea of a reduction of the state vector.”

[51]

When combined with knowledgeable measurements, the cupolet-stabilizing control scheme could aid in state preparation for experiments. Cupolets are generated regardless of the current or initial state of the system, which means that if chaotic control methods are designed to stabilize cupolets from physical systems, then two systems could be synchronized to be in the same state prior to making experimental measurements. In other words, chaotic entanglement could allow for experimenters to probe further into the classical–quantum transition without interrupting an entanglement state.

### 4.7. Entropy

In quantum mechanics, entropy is used to assess the strength of an entanglement [5]. In classical systems, entropy is a quantity that has been long associated with thermodynamics given that it measures statistical uncertainty. However, entropy is now understood to be deeply related to information theory because it is used to quantify the rate at which evolving classical systems generate information over time. Information in chaotic systems is typically encoded in their symbolic dynamics, in which case entropy specifically measures the growth rate of new symbol sequences as they are produced by an evolving system. The visitation sequences of the double scroll system are an example of such a symbolic dynamics.

In this interpretation, entropy is used to distinguish chaotic behavior from random or (quasi–)periodic behavior. This is achieved by calculating the Kolmogorov, or metric, entropy of a classical system. Denoted by *K*, Kolmogorov entropy ranges from K=0 for periodic or quasiperiodic systems to K→∞ for random systems. In between are the chaotic trajectories for which 0<K<∞ [42,52]. This last result follows from the fact that, although the dynamics of chaotic systems are deterministic, their aperiodicity and the geometry of their attractors are such that chaotic trajectories are confined to evolving along unique and non-stochastic paths on an attractor.

Entropy is particularly relevant to chaotic entanglement. Every freely-evolving chaotic system generates symbolic information at a positive and finite rate, but the entropy decays to zero whenever the chaotic behavior is controlled to become periodic. This would occur whenever a chaotic system is directed onto a periodic or cupolet state by the control scheme described in Section 2, or whenever two interacting chaotic systems stabilize each other through entanglement. Both scenarios trigger the collapse of chaotic to periodic behavior, during which time the entropy decreases to zero. Chaotic entanglement and stabilization are thus entropy-reversing events.

Entropy-reversing events are unusual in classical mechanics given that the Second Law of Thermodynamics states that the entropy of any closed or isolated system is never decreasing. However, in its information interpretation, decreasing entropy is not unusual because this would occur anytime periodic orbits are stabilized from chaotic systems. As mentioned previously, chaotic entanglement is so far established only at the information-theoretic stage, but it is compelling to envision the implications of detecting chaotic entanglement in physical systems. If physical systems are found to interact in this manner, then their interaction would coincide with a decrease in the (information) entropy and would potentially induce the systems into chaotic entanglement.

### 4.8. Differences with Quantum Entanglement

We have discussed several properties of chaotic systems that evoke parallels between chaotic and quantum entanglement. It is just as interesting to discuss their differences. First, superposition in a purely quantum sense refers to linear combinations of state vectors that collectively describe the state of a quantum system and that satisfy the Schrödinger equation. Conventional superposition is not supported in chaotic systems due to their nonlinear governing equations. Superposition instead refers to a chaotic system existing as a mixture of its cupolets, or more precisely, its UPOs. In this framework, the state of a chaotic system is well-represented as a linear combination of the states of these periodic orbits. As the chaotic system evolves in time, so too does its state vector because each $\alpha_{k}$ in Equation (8) represents the contribution of an associated cupolet to the current state of the chaotic system.

A second difference between quantum and chaotic entanglement concerns how expectedly entanglement can arise. Quantum entanglement is typically created deliberately between subatomic particles via direct interactions like atomic cascades or by spontaneous parametric down-conversions [53,54]. Interaction is also required for chaotic entanglement to arise because two chaotic systems must interactively communicate control information to each other, or the physical analog of control information. However, chaotic entanglement can also form naturally and without the aid of external preparation or control. As discussed in Section 3.3, this is known as pure entanglement, and it arises because evolving chaotic systems are constantly visiting neighborhoods of their periodic orbits. This increases the likelihood that two interacting chaotic systems are concurrently shadowing periodic orbits that could induce entanglement.

Third, unlike quantum entanglement, which allows for spontaneous nonlocal correlations, chaotic entanglement is neither distance-independent nor instantaneous in its response to measurements. Although chaotic entanglement currently exists strictly at the information-theoretic stage, there is much potential for it to manifest in physical systems. Either these physical systems must be evolving in close proximity, or there must be a channel available for information exchange in order for their communication to induce an entanglement. Should two chaotically-entangled systems become spatially separated, or simply lose the ability to communicate, then the efficacy of their interaction would diminish to zero, leading to a loss of entanglement. Each system would transition to chaotic behavior because their trajectories are no longer being directed along periodic orbits. Unlike what transpires in quantum entanglement, this transition would not be instantaneous. The previously-entangled systems would continue to evolve in close proximity of its cupolet for a period of time proportional to the cupolet’s local Lyapunov exponent. As a consequence, chaotic entanglement does not exhibit instantaneous action at a distance.

This delayed response to measurement would allow for entanglement to be reacquired between two previously-entangled chaotic systems, provided that the correct control sequences are reinstated. If the interaction is restored quickly enough between the two systems, either by shortening their spatial separation or by removing any communication barriers, then the two systems would not have drifted too far from their previously-stabilized periodic orbits. With their interaction reinstated, the two systems would redirect each other back onto their respective periodic orbits and resume their entanglement.

To summarize, the key ingredients missing from our list of classical analogs to quantum mechanical characteristics are superposition in a conventional sense, nonlocality, and the instantaneous response to measurement. In quantum mechanics, when a measurement is applied to one of two entangled particles, the state vectors of both particles each instantly collapse onto a specific state vector regardless of spatial separation. Chaotic entanglement, in contrast, is limited by physical distances, and it exhibits delayed and resilient responses to measurement due to the influence that UPOs have on the dynamics of chaotic systems.

## 5. Conclusions

For several decades, it has been the goal of many mathematicians and physicists to establish connections between classical chaos and quantum physics. Some researchers have posited how nonlinearities explain the paradoxes of certain Bell inequalities that arise in quantum mechanics [13,55], while other researchers have detected chaotic behavior in true quantum settings [5]. The research that we recently documented in [25] and that we have further discussed in this paper considers the classical–quantum correspondence from a classical perspective.

Though cupolets are themselves periodic orbits that have been stabilized from a chaotic system, the parallels that chaotic entanglement evokes with quantum entanglement are worthy of consideration. As a key example, any measurement not possessing full knowledge of the cupolet control scheme would destroy the entanglement, yet detailed knowledge would allow the control information stored in entangled cupolets to be recovered without completely compromising the entanglement. Furthermore, although cupolets could not be used to rigorously formulate a conventional Hilbert space model for an associated chaotic system, the state vectors of chaotic systems are still represented as superpositions of cupolets or UPOs. In this framework, the quantum notions of measurement, entanglement, collapse of a wave function, and entropy are all supported by chaotic systems. In particular, chaotic entanglement is an entropy-reversing event.

This identification of quantum signatures in chaotic systems can be pushed a long way, only to reach a limit when nonlocality is considered. In order to detect chaotic entanglement in interacting physical systems, the interaction cannot be spatially separated beyond a communication horizon, nor can one expect the entanglement to arise instantaneously. It seems unlikely that a classical analog of nonlocality will ever be established. Even so, there is merit in examining quantum mechanics from a classical perspective. Doing so allows one to identify the features of entanglement that are quintessentially quantum mechanical and to appreciate the unique role that nonlocality plays in quantum mechanics. There may be other discrepancies as well, and it is hoped that these differences may be used to detect whether an observed entanglement is produced via a quantum process, or whether there may be underlying chaotic processes at work.

The key point is that a classical version of entanglement has been observed from among the dense set of UPOs of a typical chaotic system. This is significant because the properties of chaotic behavior actually increase the likelihood that physical systems enter into naturally entangled states without the intervention of external controls. In theory, if this occurs in a large enough ensemble of chaotic systems, the result could be a chain reaction of stabilizations that arises naturally. It would be interesting to see if the resulting lattice of entangled cupolets elicit connections to Ising models [56].

In the preliminary investigations to date, the chaotic systems are exchanging information, but because they are not yet being driven by physical forces, one future research direction would be to investigate mechanisms by which chaotic entanglement manifests in physical systems. There is therefore much potential for cross-fertilization of this work with other research areas since many of the exchange functions in [25] are inspired by documented studies of interacting physical systems. Consequently, we are investigating certain Hamiltonian systems that are known to be chaotic, as well as several physical systems where an interaction is defined through a short-range force. Just as interesting is the possibility that chaotic entanglement may be achievable using entirely new materials, whereby the chaotic properties are found at the molecular or atomic level. Indications of such chaotic behavior have already been documented in [57,58,59]. If such entanglement can be found and manipulated, then opportunities would exist for developing new technologies.

It is hoped that the discussion presented here and in [25] will motivate the derivation of additional exchange functions with direct applicability to other research areas. This would generalize our results and uncover connections between the statistical and deterministic descriptions of chaotic dynamics, which could then be used to explain the natural entanglement that arises in several physical systems. In summary, chaotic entanglement may well be a still-undiscovered property of certain physical systems, and we hope that this research will lay the groundwork for the discovery of chaotically-entangled states in the physical world.

## Figures and Tables

**Table 1:** The following table summarizes the chaotic entanglement induced between two interacting cupolets, C000011111 (of Chaotic System I) and C011101111 (of Chaotic System II). The orbits of these cupolets are depicted in Figure 3, while the generation of the entanglement via a ‘preponderance’ exchange function is illustrated in Figure 4. Notice that the control sequence required to sustain the stability of cupolet C000011111 is contributed by cupolet C011101111 via this cupolet’s emitted sequence, E000011111. Similarly, the stability of C011101111 is maintained by the repeated application of emitted sequence E011101111, which is generated by C000011111 via the same exchange function. The font colors in this table are intended to accentuate the correspondence between the cupolets’ control sequences and their emitted sequences. Details of the generation of this entanglement are found in the text.

	Cupolet	Visitation Sequence	Emitted Sequence
Chaotic System I	C000011111	V000111111	E011101111
Chaotic System II	C011101111	V000000000111111111	E000011111
